# Differentiation-Associated Downregulation of Poly(ADP-Ribose) Polymerase-1 Expression in Myoblasts Serves to Increase Their Resistance to Oxidative Stress

**DOI:** 10.1371/journal.pone.0134227

**Published:** 2015-07-28

**Authors:** Gábor Oláh, Bartosz Szczesny, Attila Brunyánszki, Isabel A. López-García, Domokos Gerö, Zsolt Radák, Csaba Szabo

**Affiliations:** 1 Department of Anesthesiology, The University of Texas Medical Branch, Galveston, TX, United States of America; 2 Faculty of Physical Education and Sport Sciences, Semmelweis University, Alkotás Str. 44, Budapest, Hungary; 3 Shriners Hospital for Children, Galveston, TX, United States of America; Johns Hopkins University, UNITED STATES

## Abstract

Poly(ADP-ribose) polymerase 1 (PARP-1), the major isoform of the poly (ADP-ribose) polymerase family, is a constitutive nuclear and mitochondrial protein with well-recognized roles in various essential cellular functions such as DNA repair, signal transduction, apoptosis, as well as in a variety of pathophysiological conditions including sepsis, diabetes and cancer. Activation of PARP-1 in response to oxidative stress catalyzes the covalent attachment of the poly (ADP-ribose) (PAR) groups on itself and other acceptor proteins, utilizing NAD^+^ as a substrate. Overactivation of PARP-1 depletes intracellular NAD^+^ influencing mitochondrial electron transport, cellular ATP generation and, if persistent, can result in necrotic cell death. Due to their high metabolic activity, skeletal muscle cells are particularly exposed to constant oxidative stress insults. In this study, we investigated the role of PARP-1 in a well-defined model of murine skeletal muscle differentiation (C2C12) and compare the responses to oxidative stress of undifferentiated myoblasts and differentiated myotubes. We observed a marked reduction of PARP-1 expression as myoblasts differentiated into myotubes. This alteration correlated with an increased resistance to oxidative stress of the myotubes, as measured by MTT and LDH assays. Mitochondrial function, assessed by measuring mitochondrial membrane potential, was preserved under oxidative stress in myotubes compared to myoblasts. Moreover, basal respiration, ATP synthesis, and the maximal respiratory capacity of mitochondria were higher in myotubes than in myoblasts. Inhibition of the catalytic activity of PARP-1 by PJ34 (a phenanthridinone PARP inhibitor) exerted greater protective effects in undifferentiated myoblasts than in differentiated myotubes. The above observations in C2C12 cells were also confirmed in a rat-derived skeletal muscle cell line (L6). Forced overexpression of PARP1 in C2C12 myotubes sensitized the cells to oxidant-induced injury. Taken together, our data indicate that the reduction of PARP-1 expression during the process of the skeletal muscle differentiation serves as a protective mechanism to maintain the cellular functions of skeletal muscle during oxidative stress.

## Introduction

Poly(ADP-ribose) polymerase 1 (PARP-1), the major member of the PARP family, is a constitutive nuclear and mitochondrial enzyme that plays important roles in DNA repair, gene transcription, and chromatin remodeling [1–4]. It also plays a critical role in modulating cellular conditions via posttranslational modification of proteins through poly-ADP-ribosylation (PARylation), which is the addition of PAR adducts to target proteins including itself. PARP-1 has also been associated with shifting intracellular energetic pools and regulating cellular bioenergetics [1–4]. DNA damage induced by oxidative or nitrosative stress results in activation of PARP-1 [1–4]. Overactivation of PARP-1 can deplete intracellular NAD^+^ pools leading to an impairment of cellular bioenergetic homeostasis and necrosis [1–4].

Skeletal muscles account for approximately 35–40% of human body weight and are composed of striated muscle tissue. Adult muscle is a relatively stable metabolic tissue under resting conditions, but its oxygen consumption markedly increases during exercise. Under these conditions (as well as in various pathophysiological conditions and during physiological aging), intracellular production of oxidative free radicals is enhanced, mainly due to the leakage of superoxide from the mitochondrial electron transport chain [5–8]. Therefore, it is essential that skeletal muscle develops appropriate protective mechanisms to defend itself from repetitive bursts of oxidative stress; a diverse range of defensive mechanisms have been described in this respect including increase of 8-OHdG repair, higher activity of antioxidant enzymes, and changes in DNA base excision repair capacity, to name a few [9–13]. Additionally, skeletal muscle has an ability to regenerate from satellite cells (skeletal muscle-specific progenitor cells) [14–16]. Myogenic differentiation is a highly orchestrated sequence of events that produces mature skeletal muscle. Very often this process is induced by muscle injury (e.g. caused by extensive exercise), or by other pathophysiological conditions that leads to muscle loss, e.g. in patients with muscular dystrophy, advanced cancer, AIDS or burn [17–20]. Satellite cells can re-enter the cell cycle and, after proliferation, irreversibly withdraw from the cell cycle, differentiate, and with existing myofibrils to form muscle fiber [21–23]

The C2C12 cell line is widely used as a cellular model to study the process of skeletal muscle differentiation [24–27]. We have recently observed that the mitochondrial DNA of myoblasts is particularly sensitive to oxidative stress mainly due to low expression of critical mitochondrial DNA repair-specific enzyme [28]. In the current study we investigated the expression of PARP-1 in C2C12 myoblasts and myotubes in connection with oxidative stress outcomes in both cell types. Key observations were also confirmed in a second cell line (L6). The results of this study suggest that downregulation of PARP-1 expression in myoblasts correlates with enhanced resistance to oxidative stress in differentiated myotubes.

## Materials and Methods

### Reagents

Unless otherwise indicated, all reagents were purchased from Sigma–Aldrich (St. Louis, MO, USA). Fetal bovine serum (FBS), horse serum, and 0.25% trypsin-EDTA were purchased from Life Technologies (Carlsbad, CA, USA).

### Cell culture

The murine C2C12 (Catalog# ATCC CRL-1772) and rat L6 (Catalog#ATCC CRL1458) skeletal muscle cell lines were purchased from the American Type Culture Collection (ATCC, Manassas, VA, USA). Undifferentiated, proliferating C2C12 and L6 myoblasts were cultured in DMEM (ATCC, Cat#30–2002) containing 15% and 10% FBS, respectively. Differentiation for both cell lines was induced by changing the culture medium to DMEM containing 2% horse serum [16,28], **50**). All cells were maintained at 37°C, 5% CO_2_. In supporting experiments we also used the human monocyte histiocytic lymphoma cell line, U937 (ATCC CRL-1593.2). Differentiation of U937 cells was induced by incubating cells with 150 nM phorbol 12-myristate 13-acetate (PMA) for 4 days [17].

### Preparation of whole-cell extracts and Western blots

Whole-cell extracts were prepared using NP-40 lysis buffer (20 mM Tris–HCl, pH 8.8, 100 mM NaCl, 1 mM EDTA, 0.5% Nonidet P-40, 12 mM Na-deoxycholate). Cell homogenates were incubated for 30 min on ice followed by a clean-up centrifugation at 20,000 × g for 10 min at 4°C. Protein concentration was determined with Pierce BCA Protein Assay Reagent (Thermo Scientific) using bovine serum albumin as a standard. Proteins were separated by SDS-PAGE and transferred to a nitrocellulose (Bio-Rad) membrane. The membrane was blocked with StartingBlock Blocking Buffer (Thermo Scientific) for 1 h followed by incubation with primary antibody: PARP-1 (1:1,000; Cell Signaling, Cat#9532), PAX7 (1:1,000; Abcam, Cat#ab34360), myogenin (1:1,000; Abcam, ab124800), PCNA (1:1,000; Cell Signaling, Cat#2586), Histone H3 (1:1,000; Cell Signaling, Cat#12648P), ATP synthase (subunit alpha) (1:1000, Life Technologies, Cat#459240/G0531), actin-HRP (1:5,000; Santa Cruz Biotechnology, Cat#sc-1616 HRP); followed by incubation with anti-mouse or anti-rabbit secondary antibodies (Cell Signaling). The membrane was developed with SuperSignal West Pico Chemiluminescent Substrate (Pierce) and visualized in a GeneBox Detection System (Syngene).

### MTT viability assay

The MTT assay was performed as described earlier [29]. Briefly, cells were incubated with MTT reagent at a final concentration of 0.5 mg/ml, for 1 h at 37°C. The cells were washed with PBS and the residual formazan was dissolved in DMSO; subsequently, absorbance was measured at 570 nm with a background correction at 690 nm using a Molecular Devices M2 microplate reader.

### LDH cytotoxicity assay

Lactate dehydrogenase (LDH) release was measured as described previously [30]. Briefly, 30 μl of supernatant from cultured cells was mixed with 100 μl of freshly prepared LDH assay reagent containing 85 mM lactic acid, 1 mM nicotinamide adenine dinucleotide (NAD^+^), 0.27 mM N-methylphenazonium methyl sulfate (PMS), 0.528 mM INT, and 200 mM Tris (pH 8.2). Changes in absorbance were measured kinetically at 492 nm for 15 min, 37°C, using a monochromator-based reader (Powerwave HT, Biotek).

### Measurement of NAD^+^ levels

Total cellular NAD^+^ was determined using NAD^+^/NADH Cell-Based Assay Kit (Cayman Chemical, Ann Arbor, MI, USA) according to manufacture’s recommendations and as previously described [31]. The amount of formazan produced, which is proportional to the amount of NAD^+^ in the cell lysate, was measured at 450nm with SpectraMax M2 microplate reader (Molecular Devices Corp., Sunnyvale, CA, USA).

### Annexin V-phycoerythrin (Annexin V-PE) -7-aminoactinomycin D (7-AAD) staining for apoptosis/necrosis detection by flow cytometry

Detection of cell death was performed using PE Annexin V Apoptosis Detection Kit I (BD Biosciences Pharmingen, San Diego, CA) according to the manufacturer’s recommendations. Briefly, control and treated cells were trypsinized, washed in ice-cold PBS and re-suspended in 1 ml Binding Buffer. 1×10^5^ cells in 500 μl were incubated with PE Annexin V and 7-AAD for 10 min at 25°C in the dark, and analyzed immediately using a Guava EasyCyte Plus Flow Cytometer (Millipore, Billerica, MA). CytoSoft 5.3 software was used to estimate the subpopulations of early and late apoptotic, as well as necrotic cells, as a percentage of the total cell count [32].

### Bioenergetic analysis in isolated mitochondria

The XF24 Extracellular Flux Analyzer (Seahorse Biosciences, North Billerica, MA) was used to measure mitochondrial bioenergetic function. Mitochondria were isolated and extracellular flux analysis was performed as previously described [33–35]. Respiration by the mitochondria (7.5 μg/well) was sequentially measured in a coupled state with substrate present (5.5mM succinate; basal respiration, State 2), followed by State 3 (phosphorylating respiration, in the presence of ADP and substrate), State 4 (non-phosphorylating or resting respiration) following conversion of ADP to ATP, and State 4o, induced with the addition of oligomycin. Next, maximal uncoupler-stimulated respiration (State 3u) was detected by the administration of the uncoupling agent FCCP. At the end of the experiment the Complex III inhibitor, antimycin A, was applied to completely inhibit mitochondrial respiration. Inclusion of rotenone with succinate in the initial condition (State 2) triggers the respiration to be driven only by Complex II–IV. This ‘coupling assay’ examines the degree of coupling between the electron transport chain (ETC) and the oxidative phosphorylation (OXPHOS), and can distinguish between ETC and OXPHOS with respect to mitochondrial function/dysfunction.

### Mitochondrial membrane potential assay

Changes in mitochondrial membrane potential were monitored with TMRE Membrane Potential Kit from Life Technologies (Carlsbad, California, USA) according to manufacturer's instructions and as previously described [33]. Briefly, myoblasts were seeded at a concentration of 1×10^4^ cells per well in 96-well plates. For differentiated myoblasts, after the initial 24 h incubation, the culture medium was replaced with differentiation media and incubated for an additional 5 days. Next, cells were exposed to various concentrations of H_2_O_2_ for 24h. 50 nM TMRE was added to the media and cells were incubated for an additional 20 min at 37°C, 5% CO_2_; 10 and 30 μM FCCP were used as positive controls. Changes in fluorescence (ex549/em575) were monitored by monochromator-based reader (Powerwave HT, Biotek).

### Fluorescence microscopy

Myoblasts and myotubes were fixed with 4% paraformaldehyde in PBS at room temperature for 15 min, washed with PBS, and permeabilized with 0.5% Triton X-100 in PBS for 15 min at 21°C. After washing with PBS, coverslips were incubated first in 0.5% Triton X-100 containing 2.5% horse serum in PBS for 30 min, and then with primary antibodies overnight at 4°C. For the detection of PARP-1, an anti-PARP-1 antibody (Genetex, Cat#GTX61031) was used followed by Alexa Fluor 546 anti-rabbit antibody (Life Technologies, Cat#A11035). For myogenin detection, anti-myogenin antibody (Abcam, ab124800) was used followed by Alexa Fluor 488 anti-rabbit antibody (Life Technologies, Cat#A11034). Cells were washed three times with PBS and fluorescence was visualized using a Nikon Eclipse 80i inverted microscope with a Photometric CoolSNAP HQ2 camera and the NIS-Elements BR 3.10 software (Nikon Instruments, Melville, NY, USA).

### PARP-1 silencing by small-interfering RNA and bioenergetic analysis in PARP-1 silenced cells

Cells (1×10^5^ /well) were seeded into 6-well tissue culture treated plates and cultured to reach approximately 50% confluence in 24h. Next, cells were transfected with 40 nM PARP-1 specific siRNA (cat#4390771 s62054, Applied Biosystems/Ambion, Austin, TX, USA) using Lipofectamine 2000 (Invitrogen, Carlsbad, CA, USA) according to manufacture’s recommendations. Scrambled siRNA (Ambion, Silencer Negative Control#1) was used as a control. After 24h, cells were harvested and seeded onto 24-well XF24 cell culture plates. On the following day, XF24 Extracellular Flux Analyzer was used to measure cellular bioenergetics as described **(25)**.

### Transient transfection of myoblasts with PARP1

Myoblasts were transfected on 96-well plates with full-length mouse PARP-1 cDNA inserted into pCMV6-Entry vector (Myc-DDK-tagged) purchased from Origene Technologies (Cat#MR211449) (Rockville, USA, MD). Insert free plasmid, pCMV-Entry was used as control (Origene, Cat#PS100001). Transfection of myoblasts was performed using Lipofectamine 2000 (Life Technologies), according to the manufacturer's instructions. Briefly, DNA (0.2 μg/well) and Lipofectamine 2000 (1 μl/well) were separately diluted in 25 μl of Opti-MEM (Gibco). Next, DNA was added to the Lipofectamine 2000 reagent and the lipid/DNA mixtures were allowed to form complex for 5 min at room temperature. Cells were washed once with 100 ul of PBS and 100 μl of DMEM containing 15% FBS/well was added to each well. Next, lipid/DNA mixture was added and cells were incubated at 37°C, 5% CO_2_. After 24 h, transfection medium was removed and replaced with DMEM containing 2% horse serum to start differentiation. To validate the expression of PARP-1, anti-DDK mouse monoclonal antibody (1:1,000, Origene, Cat#TA50011-100) was used. After 5 days of differentiation, differentiation was confirmed visually (methods as described above) and oxidant sensitivity of the cells was tested by exposing the cells to hydrogen peroxide (0.8 mM) followed by the measurement of LDH release into the culture medium (methods as described above).

### Statistical analysis

Data are shown as means ± SEM and SD. One-way ANOVA was applied for statistical analysis, and Tukey’s post-hoc test was used for the determination of significance between individual groups. The value of p<0.05 was considered statistically significant. All statistical calculations were performed using Graphpad Prism 5 analysis software. All experiments were performed at least 3 times on different days.

## Results

### Myoblast differentiation is associated with downregulation of PARP-1 expression

**Fig 1:**
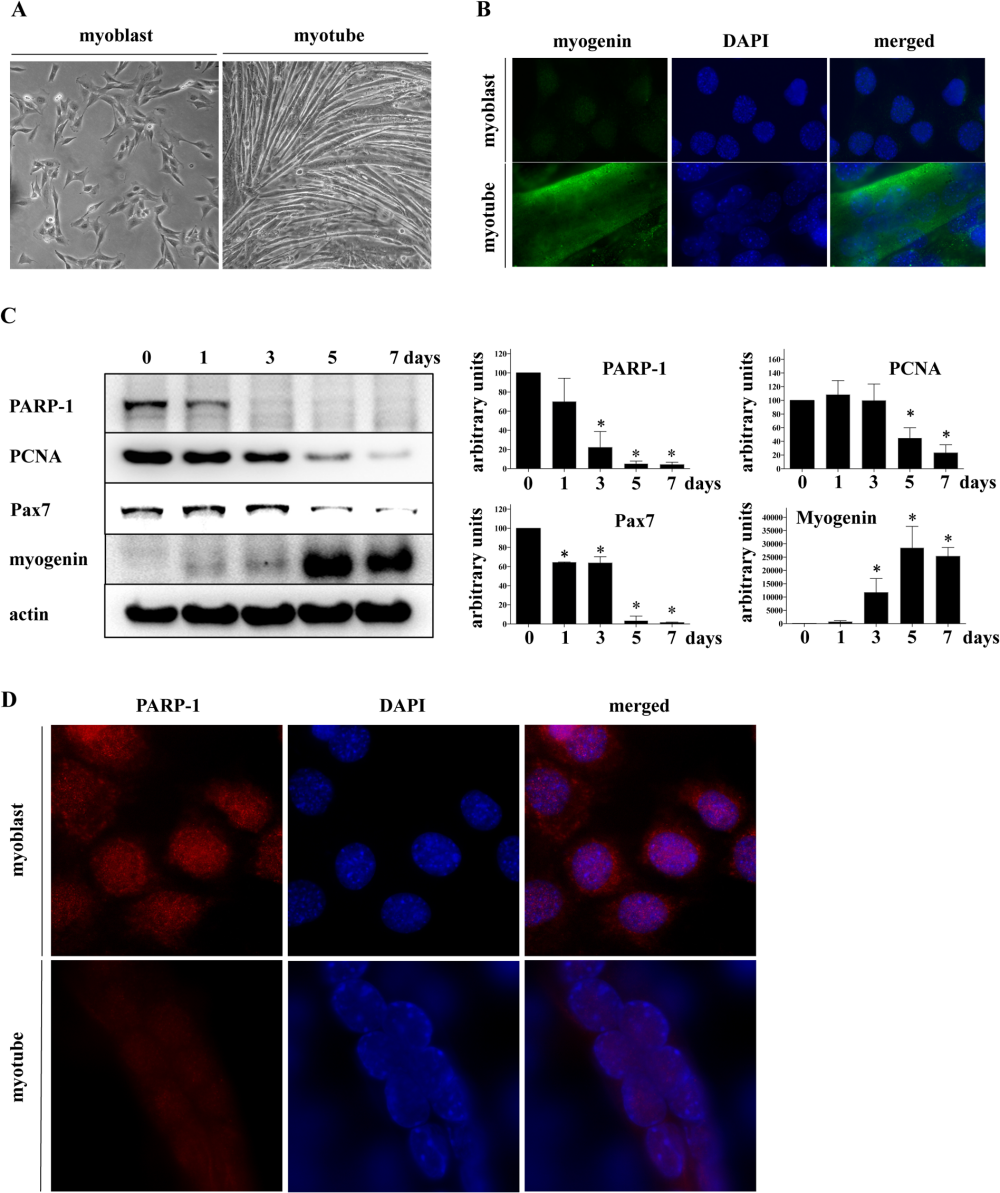
PARP1 level is reduced in myotubes. **(A)** Representative phase-contrast microscopy images of myoblasts and myotubes showing typical morphological differences. (**B**) Immunocytochemistry of the differentiation marker, myogenin, in myoblasts and myotubes. DAPI was used for nuclear counterstaining. Increased myogenin signal was detectable in the fully differentiated myotubes. **(C)** The effect of differentiation on protein expression of PARP-1 and the differentiation markers, PCNA, Pax7, and myogenin were monitored on Days 0–7 using whole cell extracts. Actin was used as a loading control. The relative quantity of proteins was calculated by densitometry and normalized to actin based on the analysis of three independent Western blots. * indicates *p<*0.05 relative to myoblasts at Day 0 (100%). (**D)** PARP-1 distribution in undifferentiated myoblasts and differentiated myotubes. DAPI was used for nuclear counterstaining. Decreased PARP-1 signal was detectable in the fully differentiated myotubes.

**Fig 2:**
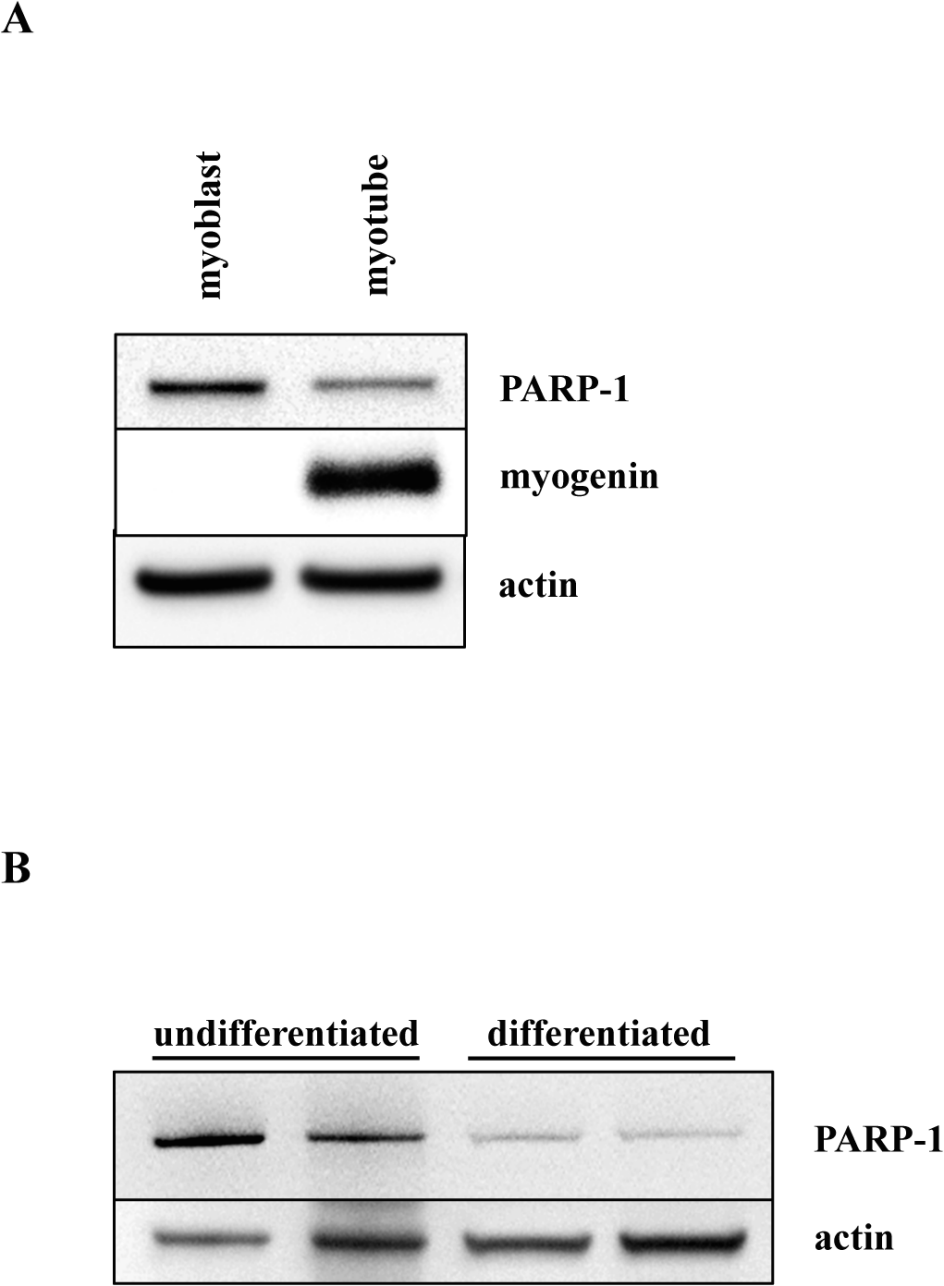
Expression of PARP-1 is reduced in differentiated L6 and U937 cells. **(A)** Western blot analysis of PARP-1, myogenin as a differentiation marker, and actin show the relative quantity of PARP-1 and the differentiation of L6 myoblasts and myotubes. **(B)** Western blot analysis of PARP-1 and actin as loading control in undifferentiated and differentiated U937.

The C2C12 cell line is a well-defined model for skeletal muscle differentiation that recapitulates the *in vivo* process through irreversible withdrawal from the cell cycle, repression of proliferation-associated genes, and expression of terminally differentiated muscle-specific genes [24–26]. Proliferating myoblasts differ from terminally differentiated, non-proliferating myotubes in morphology and protein expression profiles **(Fig 1A and 1B**). To confirm proper differentiation, we monitored the expression of transcription factor paired-box 7 (Pax7), proliferating cell nuclear antigen (PCNA), which is known to be inhibited during myoblast differentiation or repression of cellular proliferation, [34,35] and myogenin, which is known to be expressed in differentiated myotubes [23]. Time-course Western blot analysis of myoblast differentiation, from day 0 through 7, is shown in **Fig 1C**. These results confirm that the process of myoblast differentiation is accurately recapitulated, as shown by decreased expression of Pax7 and PCNA, and increased expression of myogenin. Moreover, we observed a marked decrease in PARP-1 expression in myotubes **(Fig 1C)**. PARP-1 expression was ten-fold greater in myoblasts than in myotubes **(Fig 1C)**. Immunocytochemical analysis showed that in myoblasts, PARP-1 is localized mostly in the nucleus with little cytoplasmic staining, whereas terminally differentiated myotubes showed a global reduction of signal intensity **(Fig 1D)**. To confirm our observation that skeletal muscle cell differentiation is accompanied by reduction in PARP-1 expression, we performed similar Western blot analyses using another well-defined model of skeletal muscle differentiation, namely, rat-derived L6 cells [16]. The obtained data clearly indicate that myotubes of L6 cells have reduced expression of PARP-1 (**Fig 2A**). Moreover, differentiation of U937 cells, induced by PMA, also showed a reduction in PARP1 expression (**Fig 2B**).

**Fig 3:**
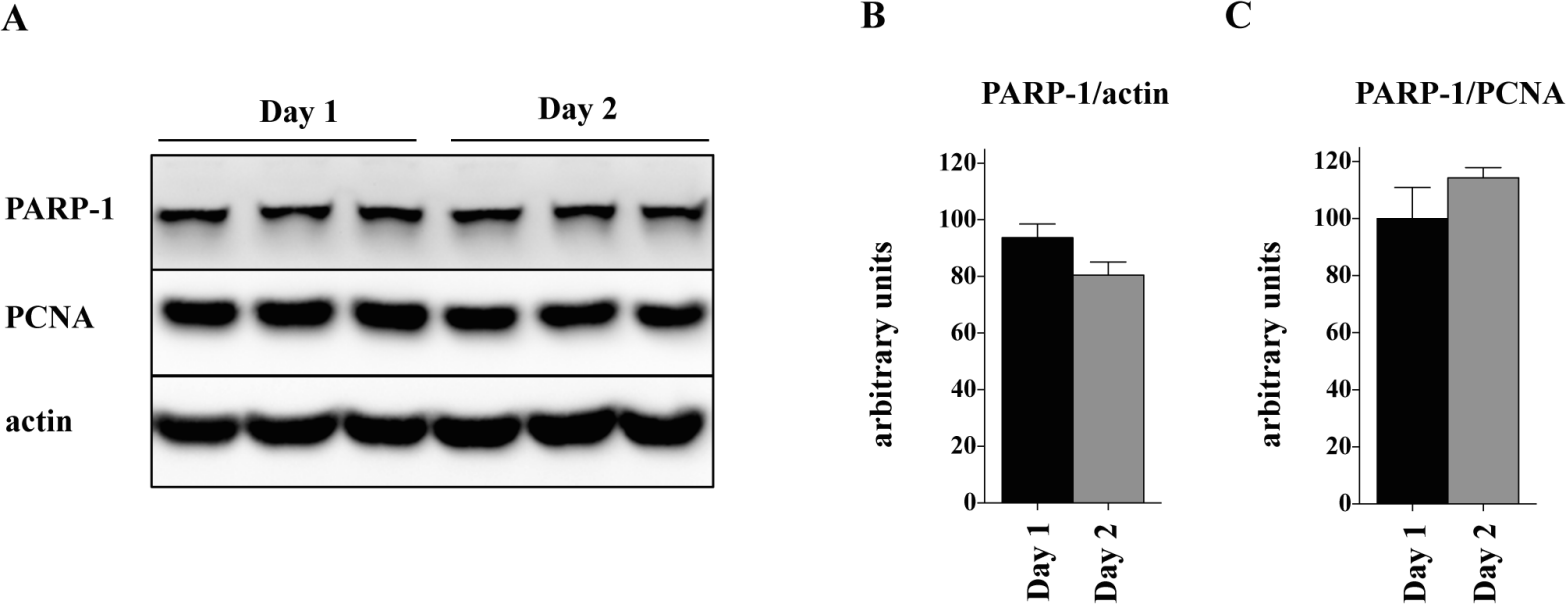
Lack of the contact inhibitory effect on PARP-1 expression in myoblasts. **(A)** Western blot analysis of PARP-1 in myoblasts at day 1 and 2 after reaching 100% confluence. Actin was used as a loading control. PARP-1 densitometric analysis was normalized to actin **(B)** or PCNA **(C)**; values obtained in Day 1 cells were set as 100%. The results show no significant difference in PARP-1 protein between cells kept for 1 or 2 days after reaching 100% confluence.

In order to investigate whether PARP-1 downregulation is an effect of contact inhibition, confluent myoblast culture was maintained for an additional one or two days while the level of PARP-1 was monitored by Western blot. As shown in **Fig 3**, the level of PARP-1 was not changed under these conditions suggesting that reduction of the PARP-1 level is an effect of differentiation, not contact inhibition.

### Differentiated myotubes are more resistant to oxidative stress

**Fig 4:**
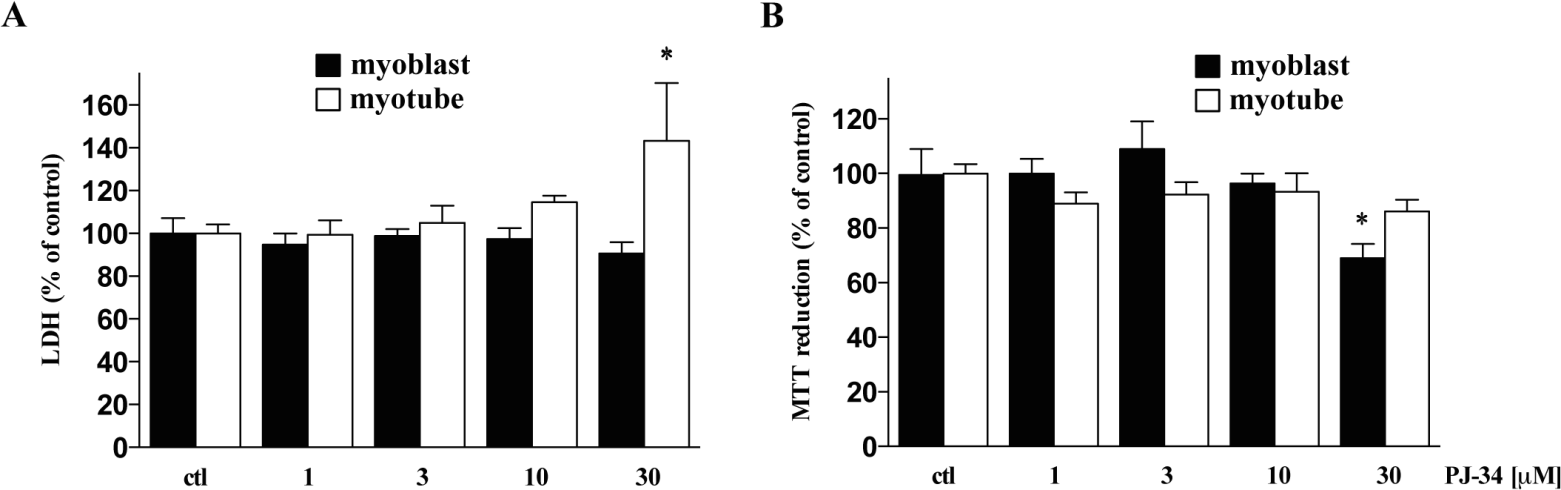
Effect of PARP-1 inhibitor, PJ34 on cell viability. Cytotoxic effect of increased concentration of PJ34 has been detected by LDH release **(A)** and MTT conversion assays **(B)**. The highest concentration of PJ34 without cytotoxic effect was 10μM. Data are shown as mean ± SD of 3 repeats. * shows significant difference, *p<*0.05, in the cell response to PJ34 relative to controls. Data shown in **A**, **B** were calculated as percentage of control cells (untreated myoblasts or myotubes, in each case set as 100%).

**Fig 5:**
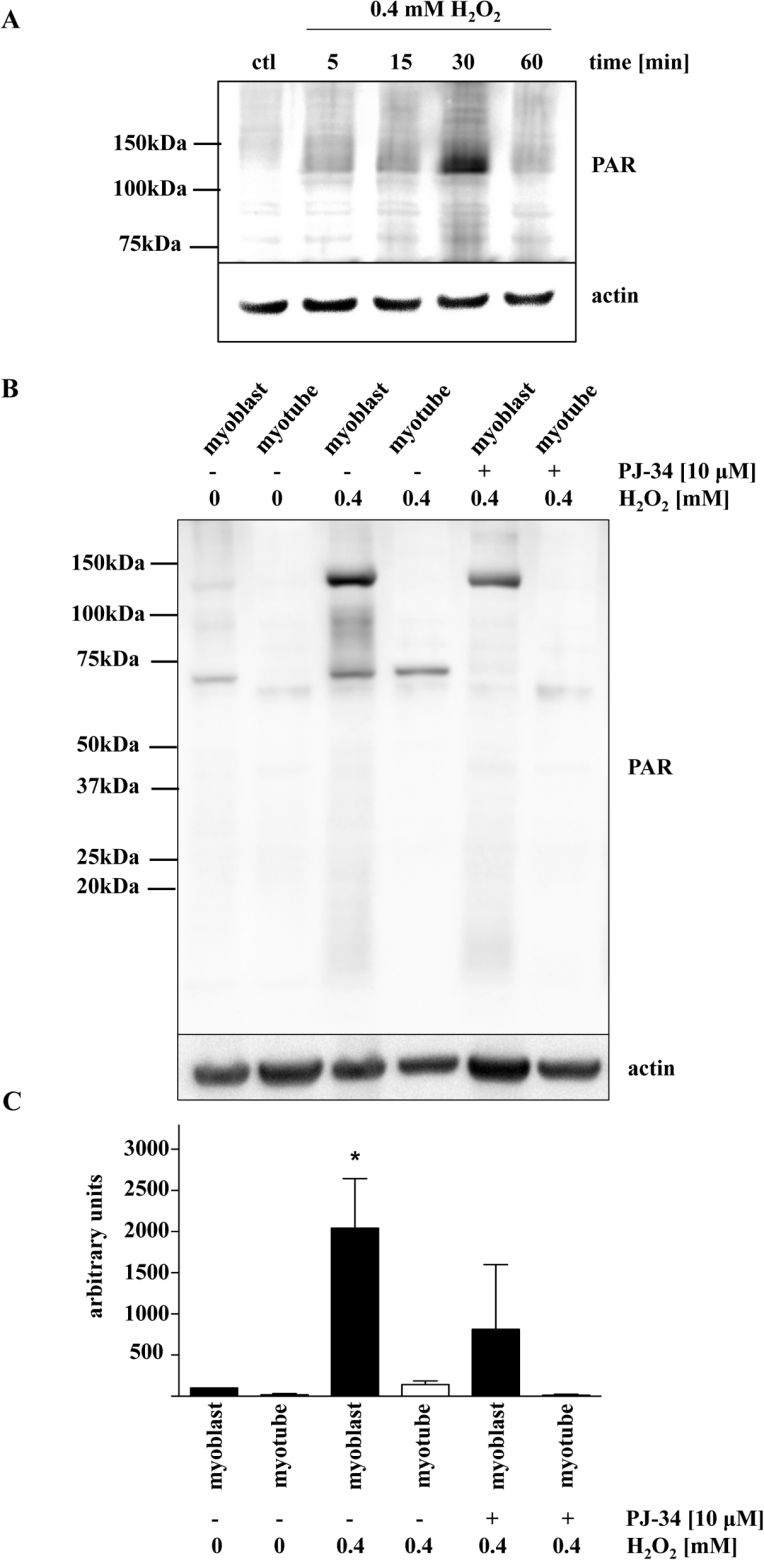
PARylation induced by oxidative stress is reduced in myotubes. **(A)** Representative Western blot shows a maximal amount of PAR signal at 30 minutes after H_2_O_2_ treatment. **(B)** Comparison of PAR formation of myoblasts and myotubes in response to exposure to 0.4 mM H_2_O_2_ in the presence or absence of PJ34 at 30 min post-treatment. **(C)** Densitometric analysis of PAR level in myoblasts and myotubes. One-way ANOVA was used for statistical analysis and determination of significance. * indicates *p<*0.05 relative to untreated myoblasts.

**Fig 6:**
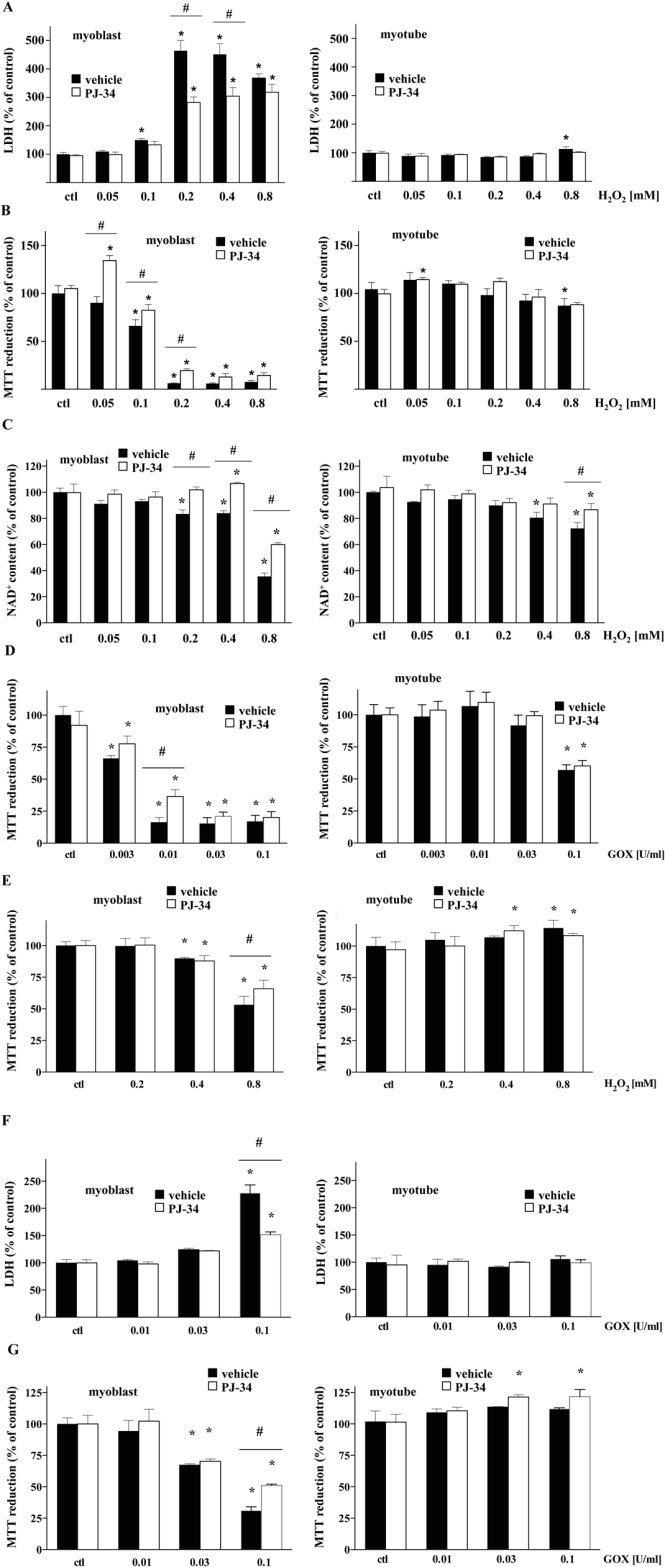
Myotubes are resistant to oxidant-induced loss of cell viability. Cell viability of myoblasts and myotubes in response to increasing concentration of oxidative stress was measured by LDH release **(A, F)**, MTT conversion **(B, D, E, G)**, and cellular NAD+ content **(C)**. 10μM PJ34 was used in all experiments to test the effect of PARP1 inhibition. Experiments shown in panels **A-D** were performed with C2C12, and those in panels **E-G,** with L6 cells. Data represent mean ± SD. One-way ANOVA was used for statistical analysis and for the determination of significance between individual groups. * shows significant difference, *p<*0.05, in the cell response to H_2_O_2_ or GOx relative to controls while # shows significant protective effect of PJ34 in H_2_O_2_ or GOx treated cells, *p<*0.05. Data shown in **A**, **B**, **C**, **D**, **E**, **F**, and **G** were calculated as percentage of control cells (untreated myoblasts or myotubes, in each case set as 100%).

In order to study the effect of PARP-1 inhibition, we first determined the maximum non-toxic concentration of well-known PARP inhibitor, PJ34 [36]. For our subsequent studies, we selected 10μM as the highest, non-toxic concentration of PJ34 based on preliminary studies with MTT conversion and LDH release cell-viability assays **(Fig 4)**. Consistent with our observations that PARP-1 expression is downregulated in myotubes as compared to myoblasts, H_2_O_2_ insult induced a lesser degree of PARP-1 activation in myotubes than in myoblasts, as determined by Western blot analysis of PAR adducts in whole-cell extracts of each cell type **(Fig 5)**. Next, we compared the changes in the viability of myoblasts and myotubes exposed to various concentrations of H_2_O_2_ by monitoring the LDH release into the culture medium, measuring the capacity of the cells to convert MTT to formazan, and quantifying cellular NAD^+^ levels. As expected, increasing concentration of H_2_O_2_ caused an increase in LDH release **(Fig 6A)**. 200 μM H_2_O_2_ resulted in a ~5-fold increase in LDH release by myoblasts but not myotubes **(Fig 6A)**. As expected, pre-treatment with the PARP inhibitor, PJ34, significantly reduced H_2_O_2_-induced LDH release in myoblasts **(Fig 6A)**. Similarly, we observed significant reduction of both MTT conversion capability and NAD^+^ levels in myoblasts exposed to increasing concentrations of H_2_O_2_, but not in myotubes **(Fig 6B and 6C)**. Similarly, PJ34 pre-treatment attenuated the deleterious effect of H_2_O_2_ in myoblasts, with only relatively minor effects in myotubes **(Fig 6B and 6C)**. The cytotoxic effects of glucose oxidase (GOx), an alternative oxidative stressor that generates constant, low levels of H_2_O_2_ in culture media, were also attenuated in PJ34-treated myoblasts in a concentration-dependent manner (assessed by measurement of MTT reduction, **Fig 6D**). Myotubes were affected only by the highest concentration of GOx and experienced no beneficial effect from PJ34 pre-treatment **(Fig 6D)**.

To verify our observations, we performed similar sets of experiments in another type of skeletal muscle cell line, namely, rat L6 cells. Similarly to C2C12, L6 myoblasts showed a distinctively higher sensitivity to oxidants—as measured by MTT conversion and LDH release assays—that could be partially attenuated by pre-treating with PJ34 **(Fig 6E, 6F and 6G)**. Myotubes were more resistant to the same concentrations of H_2_O_2_ **(Fig 6E)** and GOx **(Fig 6F and 6G)** regardless of PJ34 pre-treatment, which seemed to have no beneficial effect for myotubes.

**Fig 7:**
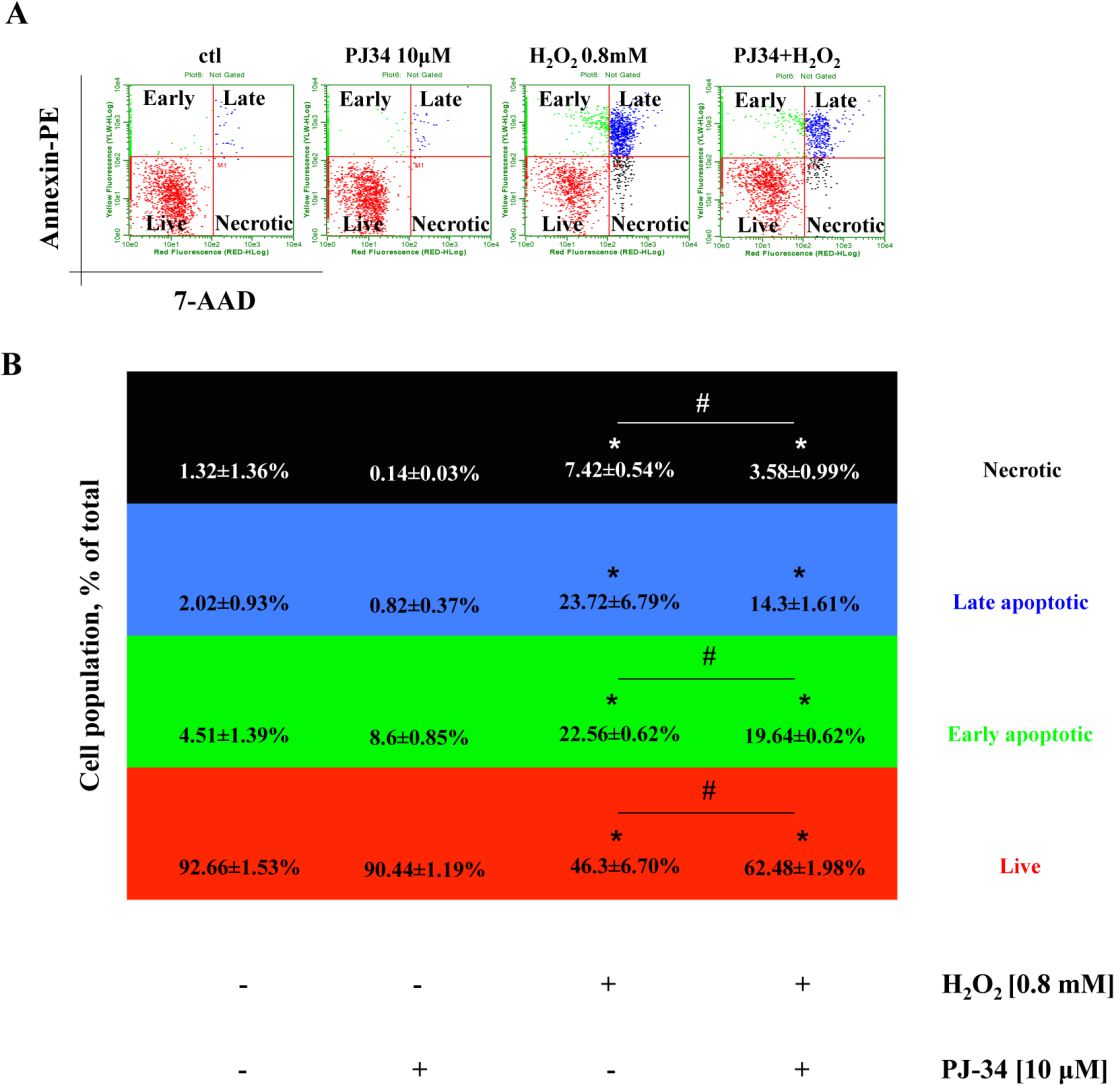
Inhibition of PARP-1 reduces subpopulations of apoptotic and necrotic cells induced by H_2_O_2_. C2C12 myoblasts were exposed to 0.8 mM H_2_O_2_ for 24h in the presence or absence of the PARP inhibitor PJ34 (10 μM). PJ34 reduced the oxidant-induced death of the myoblasts by decreasing the proportion of the necrotic, early and late apoptotic cell populations. Representative dot plots **(A)** and analysis **(B)** are shown. One-way ANOVA was used for determining significance between groups. * shows significant differences, *p ≤* 0.05, in the cell response to H_2_O_2_ relative to controls, while # shows significant protective effect of PJ34, *p≤* 0.05. Data representative of 3 different determinations conducted on different experimental days are shown. (Total cell number in each group was set to 100%).

Our observation that myotubes are resistant to oxidant induced stress was further validated by flow cytometry, which showed that oxidative stress induces cell death of mixed type (necrotic and apoptotic), and that PARP inhibition with PJ34 reduces cell death primarily by decreasing the portion of necrotic and early apoptotic cell populations **(Fig 7A and 7B)**. Taken together, these data indicate that myotubes are more resistant to oxidative stress than myoblasts, and that the oxidative stress-induced cell dysfunction/cell death in myoblasts involves a significant PARP-1dependent component.

### Myotubes preserve mitochondrial functions during oxidative stress

**Fig 8:**
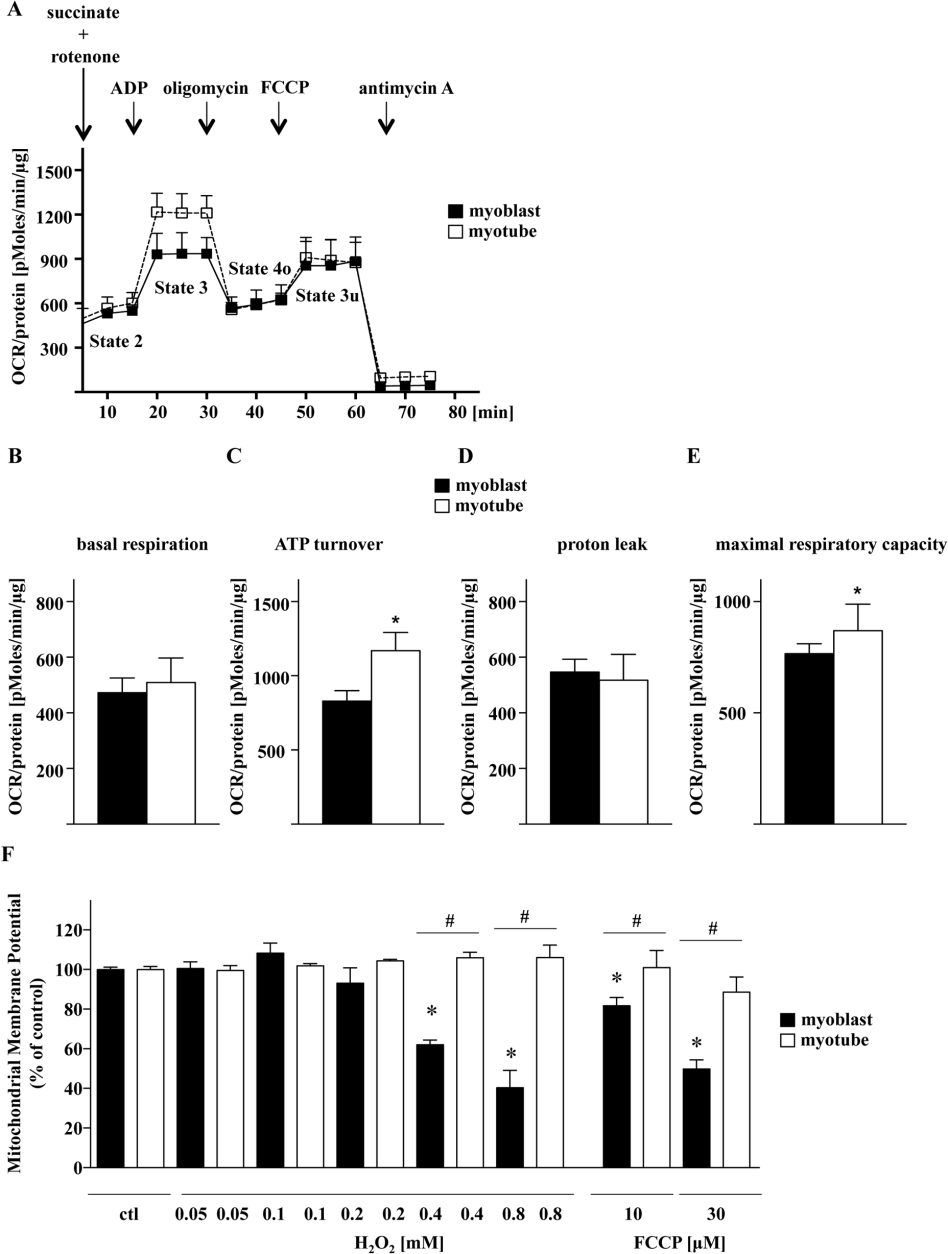
Cellular bioenergetics of mitochondria isolated from myoblasts and myotubes. Bioenergetic analysis was carried out using the Extracellular Flux Analyzer (Seahorse XF-24) in response to the sequential administration of ADP (4 mM), oligomycin (2 μM), FCCP (4 μM), and antimycin A (4 μM) in the presence of succinate (5.5 mM) and rotenone (2 μM). **(A)** Representative analysis of oxygen consumption. Calculated bioenergetics parameters: **(B)** basal respiration, **(C)** ATP turnover, **(D)** proton leak and **(E)** maximal respiratory capacity. Data are shown as means ± SD of *n* = 9 wells collected from *n* = 3 experiments performed on 3 different days. * indicates significant difference, *p <* 0.01, in ATP turnover and maximal respiratory capacity. State 2 = Basal Respiration, State 3 = ATP Turnover, State 4o = Proton Leak, State 3u = FCCP-stimulated Maximal Respiratory Capacity. **(F)** Mitochondrial membrane potential changes in the absence or presence of the PARP inhibitor PJ34 (10 μM) in C2C12 myoblasts and myotubes. Statistical analyses of n = 3 independent experiments were assessed; where * indicates significant difference, *p*<0.001, between control and H_2_O_2_-treated myoblasts, while # shows significant difference, *p*<0.001, between myoblasts and myotubes treated under the same conditions.

**Fig 9:**
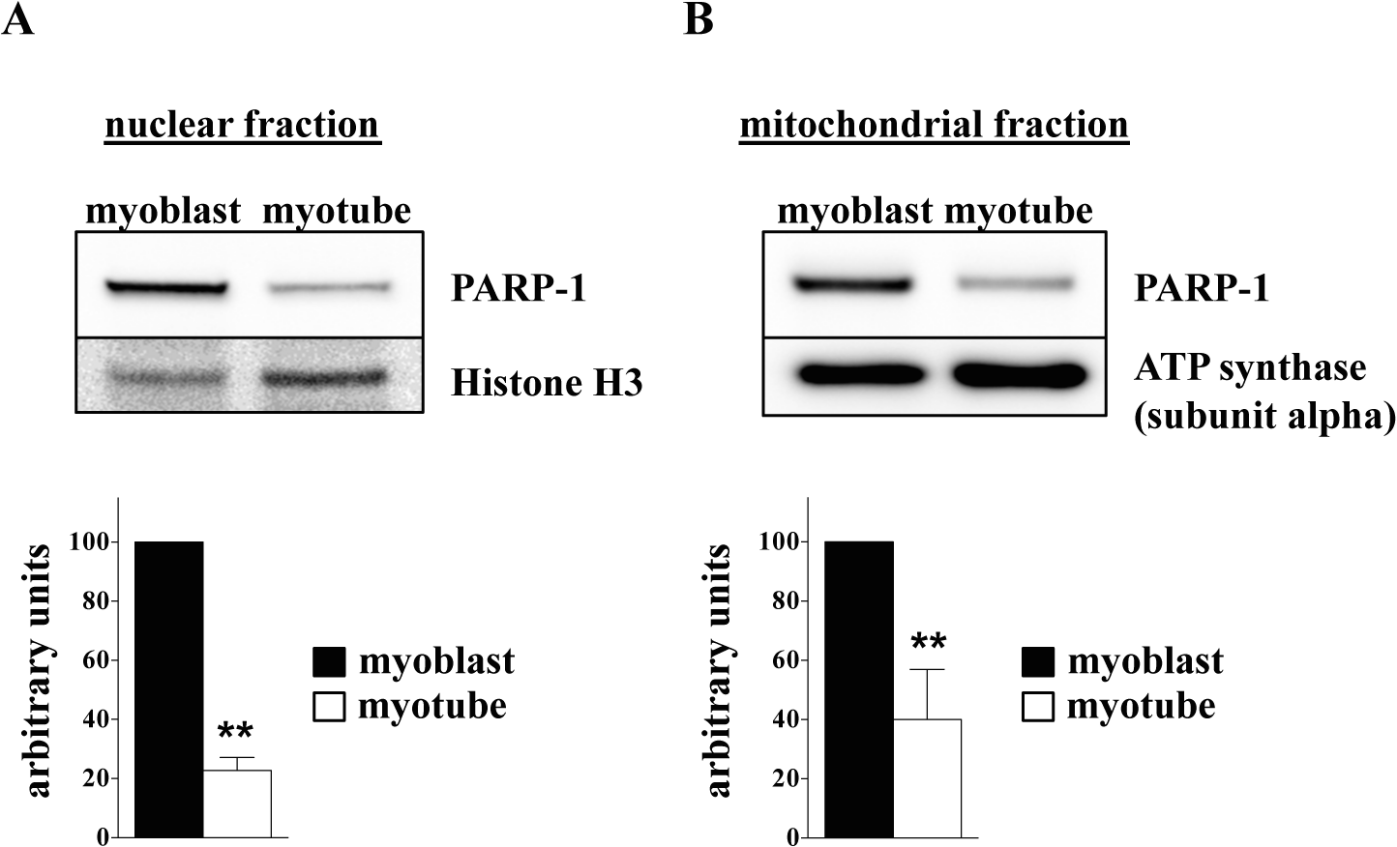
PARP-1 protein levels in mitochondrial and nuclear fractions of myoblasts and myotubes. Western blot analysis detected elevated expression of PARP-1 in myoblasts than in myotubes for both nuclear (A) and mitochondrial (B) fractions. Densitometric analysis of PARP-1 protein level in myoblast was set as 100%. Statistical analyses of n = 3 independent experiments were assessed; where ** indicates *p*<0.001.

**Fig 10:**
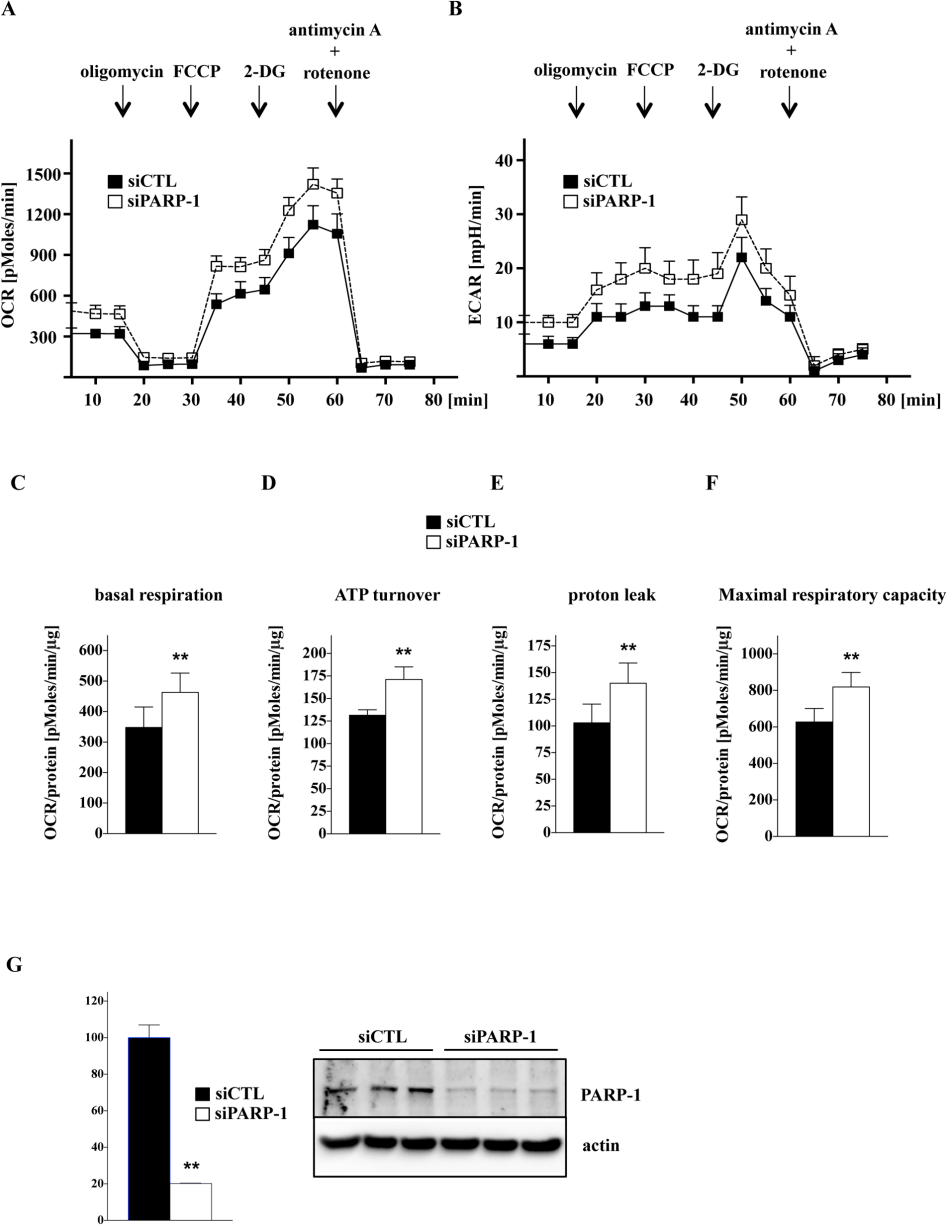
Silencing PARP-1 increases both oxidative phosphorylation and glycolytic activity of C2C12 myoblasts. Bioenergetic analysis of the myoblasts was conducted by extracellular flux analysis. In the figure, a time-course measurement of OCR (A) and ECAR (B) for 1×10^4^ cells/well under basal conditions was followed by the sequential addition of oligomycin (1 μg/ml), FCCP (0.3 μM), 2-DG (5mM) and antimycin A (2 μg/ml). Cells with PARP-1 silencing show significantly elevated OCR values during basal and uncoupled respiration (*p<0.05). **(C, D, E, F)** Major bioenergetic parameters are elevated in cells after siRNA silencing of PARP-1, compared to control as calculated by unpaired t-test (*p<0.01). Data are shown as means ± SD of *n* = 15 wells (each group) collected from *n* = 3 experiments performed on 3 different days. **(G)** Western blot analysis shows the efficiency of PARP-1 silencing as evaluated by unpaired t-test, *p < 0.001 relative to siCTL.

We investigated the differences in major bioenergetics parameters in mitochondria isolated from C2C12 myoblasts and myotubes with Extracellular Flux Analysis (**Fig 8A)**. While basal respiration (State 2) did not show any significant differences between the two cell types, myotubes were found to have higher ATP turnover (State 3) and maximal respiratory capacity (State 3u) than myoblasts. **(Fig 8B, 8C, 8D and 8E)**. To investigate the effect of H_2_O_2_ on mitochondrial functions of myoblasts and myotubes, we measured mitochondrial membrane potential. We observed a gradual decrease of the mitochondrial membrane potential in response to increasing concentration of H_2_O_2_ in the myoblasts, while the mitochondrial membrane potential was unaffected in the myotubes **(Fig 8F)**. FCCP (a mitochondrial uncoupler) was used as a control **(Fig 8F)**. Since PARP-1 was localized in the nucleus and in the mitochondria [37,38] we investigated its level in both cellular fractions. As shown in **Fig 9**, reduction of PARP-1 level was found in nuclear and mitochondrial fractions of myoblasts as compared to myotubes (**Fig 9A and 9B**, respectively). We have previously shown that PARP-1 silencing significantly enhances basal mitochondrial bioenergetics in cultured endothelial and epithelial cells [31]. Now, we investigated the effect of transient siRNA-mediated silencing of PARP-1 on the bioenergetic response in C2C12 myoblasts. Silencing PARP-1 expression increases both oxidative phosphorylation **(Fig 10A)** and glycolytic activity **(Fig 10B)**. The latter observation is in line with recent findings showing the regulation of glycolytic function by PARP-1 in neurons [39].

### Forced expression of PARP1 in myotubes increases their oxidant sensitivity

**Fig 11:**
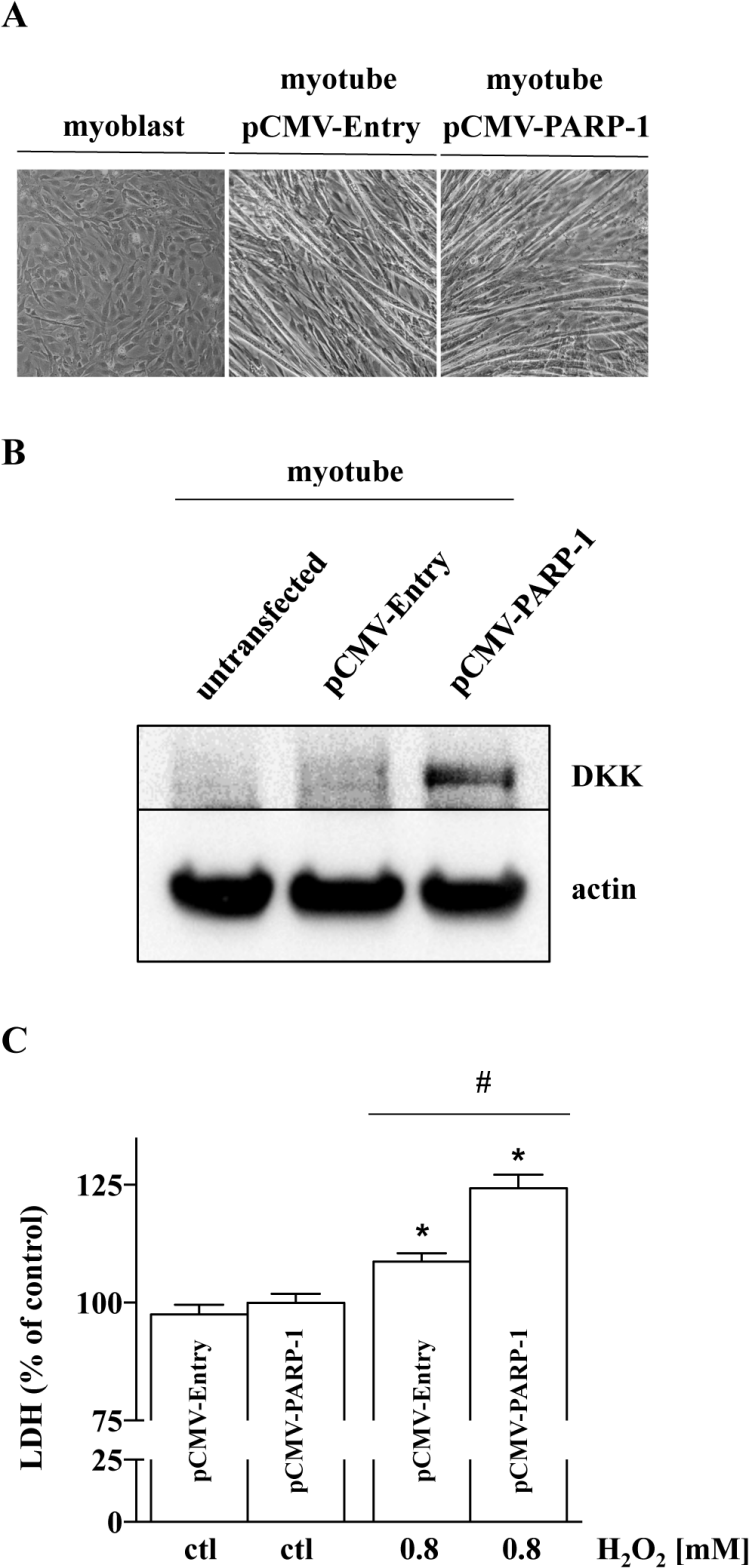
PARP-1 overexpression sensitizes C2C12 myotubes to oxidative stress. **(A)** PARP-1 overexpression does inhibit the differentiation of myoblasts to myotubes. (**B**) Representative western blot shows the presence of expressed, tagged PARP-1 in myotubes (after 5 days of differentiation from myoblasts). (**C**) Myotubes with overexpressed PARP-1 possessed increased sensitivity to oxidative stress 24 h after 0.8 mM H_2_O_2_, as estimated by the measurement of LDH release. Data represent mean ± SD of n = 6 determinations. * shows significant difference, *p<*0.05, in the cell response to H_2_O_2_ relative to controls (in the absence of H_2_O_2_), while # shows a significant increase in LDH release in the myotubes with forced PARP-1 expression, when compared to control myotubes.

There is a possibility that, in addition to the downregulation of PARP1, a host of additional alterations in the transcriptome profiles between myoblasts and myotubes resulted in the observed phenotype of decreased oxidant sensitivity in myotubes (in contrast to myoblasts). Therefore, PARP1 supplementation (forced expression of PARP1 using a cDNA inserted into pCMV6-Entry vector) was performed in myotubes, followed by differentiation of the cells into myoblasts (**Fig 11A**). The overexpressed PARP-1 maintained in myotubes as detected by Western analysis using anti-DDK monoclonal antibody (**Fig 11B**). Myotubes with forced expression of PARP1 became more sensitive to hydrogen peroxide induced cell injury: they responded with increased LDH release, compared to the sham-transfected myotube controls (**Fig 11C**).

## Discussion

The main conclusion of the present study is that protein level of PARP-1 decreases under the differentiation process of murine skeletal muscle cells, and concomitantly, the cells acquire a marked resistance to oxidative stress and develop a capacity to maintain cellular bioenergetics during oxidative challenge.

Cellular reprograming occurs during skeletal muscle differentiation with multiple genes up- and down-regulating as required to orchestrate a smooth transition from proliferation to cell cycle exit, differentiation and finally, fusion of multiple cells to form myotubes. One of the key components of the process is the expression of muscle specific transcription factors, such as myogenin, which has a primary role in down-regulating Cdk activity in order to exit the cell cycle [40]. The important role of PARP-1 in chromatin remodeling and gene expression is well recognized [1,41]. We hypothesize that maintenance of a high level of PARP-1 expression is required at the earlier stages of the differentiation process, in order to initiate the changes in the chromatin structure to facilitate reprogramming gene expression. On the other hand, the cost to the cell for this high level of PARP-1 expression is increased sensitivity to oxidative stress. Please note, however, that cells do not normally experience substantial oxidative stress in this stage of the cell's lifecycle.

The situation is quite different in fully differentiated myocytes. Significant oxidative stress is generated from normal physiological function of differentiated myocytes, e.g. skeletal muscle contraction during movement or exercise [6–13]. Therefore, we hypothesized that the downregulation of PARP-1 serves as a protective mechanism limiting the deleterious consequences of oxidant-mediated PARP-1 overactivation, such as cellular NAD^+^ depletion, decrease in bioenergetics, or, in extreme cases, cell injury. This may be particularly important for skeletal muscle, which is known to be prone to oxidative damage [5–13].

Our immunocytochemistry studies show that PARP-1 is localized mainly in the nucleus of myoblasts, but that its nuclear expression is reduced after differentiation has taken place in myotubes; this occurs parallel to an increase in perinuclear staining. Based on several lines of studies in various cell types [32,39,40], as well as data presented in the current report, this staining represents mitochondrially localized PARP-1. Relatively little is known about the functional role of mitochondrial PARP-1. Rossi and colleagues [39] showed that the mitochondrial protein mitofilin co-localized with PARP-1 in mitochondria of HeLa cells, which shows the mitochondrial presence of the enzyme. Using subcellular fractionation, we show in the current paper that myoblasts have higher levels of PARP-1 in their mitochondria, compared to the differentiated myotubes. In line with previous studies, the possible role of PARP-1 in C2C12 might be related to the higher integrity of mtDNA in the latter cells. We have recently demonstrated that there are differences in mitochondrial DNA repair capability between myoblasts and myotubes, with the myotubes having higher DNA integrity [28]. Moreover, we have recently observed that low levels of oxidative stress selectively activate mitochondrial PARP-1 in human U937 monocytes [32]. These prior findings, when taken together with the data presented in the current report, suggest that both the downregulation and the intracellular distribution of PARP-1 (from the nucleus to the mitochondria) during myoblast differentiation may contribute to a change in oxidative stress resistance. This is further supported by our recent observation that PARP-1-depletion increases the rate of the mitochondrial DNA repair [40]. Other series of studies also showed the beneficial effects of genetically disrupting and pharmacologically inhibiting PARP-1 in cerebral ischemia, oxidative and nitrosative stress in islet cells, myocardial post-ischemic injury, and cerebral artery occlusion and its effect on motor function [39–47]. To further test whether PARP-1 downregulation is an independent contributor to the resistance of myotubes to oxidative stress, PARP-1 overexpression in myotubes was also conducted; this intervention increased the oxidative stress sensitivity of the myotubes. These findings support the view that downregulation of PARP1 during differentiation of myoblasts to myotubes directly regulates the oxidant-sensitivity of these cells. Nevertheless it is obvious that a host of changes occur in the transcriptome profile during cell differentiation, and, therefore, multiple factors—in addition to PARP1 downregulation—are also likely to contribute.

There are a number of studies implicating PARP-1 in the regulation of cellular bioenergetics (under normal conditions and during oxidative stress). The deletion of PARP-1 increases NAD^+^ content and SIRT1 activity in brown adipose tissue and muscle, which mimics the responses seen with SIRT1 activation (both result in increased energy expenditure and enhanced oxidative metabolism) [48]. Moreover, in bEnd.3 endothelial and A549 epithelial cells, pharmacological inhibition or siRNA-mediating silencing of PARP-1 increased basal and uncoupled cellular respiration, suggesting that PARP-1 is a regulator of physiological mitochondrial function [31]. PARP-1^−/−^ muscle showed a marked deacetylation of PGC-1α and FOXO1, which was linked to enhanced mitochondrial biogenesis and a more oxidative profile of muscle fibers [49]. In line with the above-cited literature, the bioenergetic experiments conducted in the current study also indicate that PARP-1 is a regulator of basal mitochondrial function and suggest that PARP-1 also regulates the glycolytic capacity of myoblasts—both of these functions were enhanced when PARP-1 was silenced. Based on these data, we conclude that the downregulation and redistribution of PARP-1 during skeletal muscle differentiation will not only create an oxidative stress resistance, but it will also enhance the cellular bioenergetic capacity of the differentiated muscle cell (when compared to the same parameters of the myoblast), for instance, by consuming less NAD^+^. However, we must keep in mind that cellular NAD^+^ levels are compartmentalized and are regulated by multiple enzymes [50,51]; therefore, it is conceivable that differentiation-associated changes in the expression or activity of various enzyme systems that handle NAD^+^ (other than PARP-1) may also have contributed to the observed decrease in cellular NAD^+^ levels during differentiation. Follow-up analysis of expression level of other enzymes utilizing NAD^+^ is warranted to explain this observation. In this context, it is interesting to mention the recent report of Frederick and colleagues demonstrating that increasing NAD^+^ biosynthesis in the skeletal muscle, on its own, is not sufficient to increase metabolic function [52].

It should also be mentioned that PARP-1, while downregulated compared to myoblasts, is not completely absent in the differentiated myotubes. Accordingly, oxidative stress does have some effects on these cells, for example, in terms of NAD^+^ depletion, though these effects are relatively minor when compared to myoblasts and are reduced by PARP-1 inhibition. Also, *in vivo*, there is always a constant turnover of the muscle, meaning that the skeletal muscle probably represents a mixture of cells in various stages of differentiation (and, correspondingly, various degrees of PARP-1 expression). These notions are consistent with a recent report of Pirinen and colleagues [53], demonstrating that *in vivo*, treatment of mice with PARP-1 inhibitors exerts beneficial effects on skeletal muscle endurance and performance, coupled with increased cellular NAD^+^ content, mitochondrial function, and mitochondrial biogenesis.

Further observations made in the current study that remain to be investigated in follow-up studies include **(a)** the functional consequence of the variable regulation of the various 'minor' PARP isoforms during differentiation (i.e. downregulation of PARP-2 and PARP-7 and upregulation of PARP-3), **(b)** the mechanism of the increased glycolytic activity after PARP-1 silencing in myoblasts, and **(c)** the reasons why the downregulation of PARP-1 during differentiation is cell-type dependent. With respect to PARP-2, it is interesting to note that inhibition of PARP-2 has recently been shown to produce an increase in mitochondrial biogenesis in skeletal muscle [54].

In summary, we found that PARP-1 protein level decreases under the differentiation process of C2C12 cells. Based on multiple lines of functional data, we conclude that the functional importance of this change is that it endows myoblasts with several advantages, including oxidative stress resistance and an increased bioenergetic function. We fully realize that the regulation of PARP-1 in myoblasts and myotubes may be a specialized case since PARP-1 has multiple roles in a variety of cell types, ranging from cancer cell survival to an active factor mediating necrosis in parenchymal cells, and catalyzing pro-inflammatory gene expression in immune cells [1–3]. Changes in PARP-1 expression may well have very different roles, depending on the particular cell type and the particular (patho)physiological context.

## References

[1] Jagtap P, Szabo C (2005) Poly(ADP-ribose) polymerase and the therapeutic effects of its inhibitors. Nat Rev Drug Discov 4: 421–440. 10.1038/nrd1718 . 15864271

[2] Pacher P, Szabo C (2008) Role of the peroxynitrite-poly(ADP-ribose) polymerase pathway in human disease. Am J Pathol 173: 2–13. 10.2353/ajpath.2008.080019 . 18535182 PMC2438280

[3] De Vos M, Schreiber V, Dantzer F (2012) The diverse roles and clinical relevance of PARPs in DNA damage repair: current state of the art. Biochem Pharmacol 84: 137–146. 10.1016/j.bcp.2012.03.018 . 22469522

[4] Curtin NJ, Szabo C (2012) Therapeutic applications of PARP inhibitors: anticancer therapy and beyond. Mol Aspects Med 34: 1217–56. 10.1016/j.mam.2013.01.006 . 23370117 PMC3657315

[5] Sakellariou GK, Jackson MJ, Vasilaki A (2014) Redefining the major contributors to superoxide production in contracting skeletal muscle. The role of NAD(P)H oxidases. Free Radic Res 48: 12–29. 10.3109/10715762.2013.830718 . 23915064

[6] Hepple RT (2014) Mitochondrial involvement and impact in aging skeletal muscle. Front Aging Neurosci 6: 211 10.3389/fnagi.2014.00211 . 25309422 PMC4159998

[7] Aoi W, Naito Y, Yoshikawa T (2014) Potential role of oxidative protein modification in energy metabolism in exercise. Subcell Biochem 77: 175–87. 10.1007/978-94-007-7920-4_15 . 24374928

[8] Nikolaidis MG, Kyparos A, Spanou C, Paschalis V, Theodorou AA, Vrabas IS (2012) Redox biology of exercise: an integrative and comparative consideration of some overlooked issues. J Exp Biol 215: 1615–25. 10.1242/jeb.067470 . 22539728

[9] Radák Z, Naito H, Kaneko T, Tahara S, Nakamoto H, Takahashi R, et al (2002) Exercise training decreases DNA damage and increases DNA repair and resistance against oxidative stress of proteins in aged rat skeletal muscle. Pflugers Arch 445: 273–8. 10.1007/s00424-002-0918-6 . 12457248

[10] Ji LL (2008) Modulation of skeletal muscle antioxidant defense by exercise: Role of redox signaling. Free Radic Biol Med 44: 142–52. 10.1016/j.freeradbiomed.2007.02.031 . 18191750

[11] Radak Z, Chung HY, Goto S (2008) Systemic adaptation to oxidative challenge induced by regular exercise. Free Radic Biol Med 44: 153–9. 10.1016/j.freeradbiomed.2007.01.029 . 18191751

[12] Radak Z, Bori Z, Koltai E, Fatouros IG, Jamurtas AZ, Douroudos II, et al (2011) Age-dependent changes in 8-oxoguanine-DNA glycosylase activity are modulated by adaptive responses to physical exercise in human skeletal muscle. Free Radic Biol Med 51: 417–23. 10.1016/j.freeradbiomed.2011.04.018 . 21569841 PMC3775599

[13] Szczesny B, Tann AW, Mitra S (2010) Age-and tissue-specific changes in mitochondrial and nuclear DNA base excision repair activity in mice: susceptibility of skeletal muscles to oxidative injury. Mech Ageing Dev 131: 330–7. 10.1016/j.mad.2010.03 20363243 PMC2883317

[14] Wang YX, Dumont NA, Rudnicki MA (2014) Muscle stem cells at a glance. J Cell Sci 127: 4543–8. 10.1242/jcs.151209 . 25300792 PMC4215708

[15] Bentzinger CF, Wang YX, Dumont NA, Rudnicki MA (2013) Cellular dynamics in the muscle satellite cell niche. EMBO Rep 14: 1062–72. 10.1038/embor.2013.182 . 24232182 PMC3849491

[16] Hudson MB, Woodworth-Hobbs ME, Zheng B, Rahnert JA, Blount MA, Gooch JL, et al (2014) miR-23a is decreased during muscle atrophy by a mechanism that includes calcineurin signaling and exosome-mediated export. Am J Physiol Cell Physiol 306: C551–8. 10.1152/ajpcell.00266.2013 . 24336651 PMC3948973

[17] Stoppelli MP, Corti A, Soffientini A, Cassani G, Blasi F, Assoian RK (1985) Differentiation-enhanced binding of the amino-terminal fragment of human urokinase plasminogen activator to a specific receptor on U937 monocytes. Proc Natl Acad Sci U S A 82: 4939–43. . 2991901 10.1073/pnas.82.15.4939 PMC390473

[18] Cohen S, Nathan JA, Goldberg AL (2015) Muscle wasting in disease: molecular mechanisms and promising therapies. Nat Rev Drug Discov 14: 58–74. 10.1038/nrd4467 . 25549588

[19] Thibault R, Cano N, Pichard C (2011) Quantification of lean tissue losses during cancer and HIV infection/AIDS. Curr Opin Clin Nutr Metab Care 14: 261–7. 10.1097/MCO.0b013e3283455d60 . 21415734

[20] Diaz EC, Herndon DN, Porter C, Sidossis LS, Suman OE, Børsheim E (2014) Effects of pharmacological interventions on muscle protein synthesis and breakdown in recovery from burns. Burns pii: S0305-4179(14)00346-5. 10.1016/j.burns.2014.10.010 . 25468473 PMC4417037

[21] Hawke T, Garry D (2001) Myogenic satellite cells: physiology to molecular biology. J Appl Physiol 91: 534–551. . 11457764 10.1152/jappl.2001.91.2.534

[22] Braun T, Gautel M (2011) Transcriptional mechanisms regulating skeletal muscle differentiation, growth and homeostasis. Nature Rev Mol Cell Biol 12: 349–361. 10.1038/nrm3118 . 21602905

[23] Wang YX, Rudnicki MA (2012) Satellite cells, the engines of muscle repair. Nat Rev Mol Cell Biol 13: 127–133. 10.1038/nrm3265 . 22186952

[24] Yaffe D, Saxel O (1977) Serial passaging and differentiation of myogenic cells isolated from dystrophic mouse muscle. Nature 270: 725–7. . 563524 10.1038/270725a0

[25] Ferri P, Barbieri E, Burattini S, Guescini M, D’Emilio A, Biagiotti L, et al (2009) Expression and subcellular localization of myogenic regulatory factors during the differentiation of skeletal muscle C2C12 myoblasts. J Cell Biochem 108: 1302–17. 10.1002/jcb.22360 . 19830700

[26] Shen X, Collier JM, Hlaing M, Zhang L, Delshad EH, Bristow J, et al (2003) Genome-wide examination of myoblast cell cycle withdrawal during differentiation. Dev Dyn 226: 128–138. 10.1002/dvdy.10200 . 12508234

[27] Olguin HC, Olwin BB (2004) Pax-7 up-regulation inhibits myogenesis and cell cycle progression in satellite cells: a potential mechanism for self-renewal. Dev Biol 275: 375–388. 10.1016/j.ydbio.2004.08.015 . 15501225 PMC3322464

[28] Szczesny B, Olah G, Walker DK, Volpi E, Rasmussen BB, Mitra S, et al (2013) Deficiency in Repair of the Mitochondrial Genome Sensitizes Proliferating Myoblasts to Oxidative Damage. PLoS One 8: e75201 10.1371/journal.pone.0075201 . 24066171 PMC3774773

[29] Gerö D, Szoleczky P, Módis K, Pribis JP, Al-Abed Y, Yang H, et al (2013) Identification of pharmacological modulators of HMGB1-induced inflammatory response by cell-based screening. PLoS One 8: e65994 10.1371/journal.pone.0065994 . 23799067 PMC3682954

[30] Gerö D, Szoleczky P, Chatzianastasiou A, Papapetropoulos A, Szabo C (2014) Modulation of poly(ADP-ribose) polymerase-1 (PARP-1) mediated oxidative cell injury by ring finger protein 146 (RNF146) in cardiac myocytes. Mol Med 20: 313–328. 10.2119/molmed.2014.00102 . 24842055 PMC4153837

[31] Módis K, Gerö D, Erdélyi K, Szoleczky P, DeWitt D, Szabo C (2012) Cellular bioenergetics is regulated by PARP1 under resting conditions and during oxidative stress. Biochem Pharmacol 83: 633–43. doi: 10.1016/j.bcp.2011.12.014. 22198485 22198485 10.1016/j.bcp.2011.12.014 PMC3272837

[32] Brunyanszki A, Olah G, Coletta C, Szczesny B, Szabo C (2014) Mitochondrial poly(ADP-ribose) polymerase activation by the β-adrenoceptor/cAMP/protein kinase A axis during oxidative stress. Mol Pharmacol 86: 450–62. 10.1124/mol.114.094318 . 25069723 PMC4164979

[33] Módis K, Panopoulos P, Coletta C, Papapetropoulos A, Szabo C (2013) Hydrogen sulfide-mediated stimulation of mitochondrial electron transport involves inhibition of the mitochondrial phosphodiesterase 2A, elevation of cAMP and activation of protein kinase A. Biochem Pharmacol 86: 1311–9. 10.1016/j.bcp.2013.08.064 . 24012591

[34] Rogers GW, Brand MD, Petrosyan S, Ashok D, Elorza AA, Ferrick DA, et al (2011) High throughput microplate respiratory measurements using minimal quantities of isolated mitochondria. PLoS ONE 6: p. e21746 10.1371/journal.pone.0021746 . 21799747 PMC3143121

[35] Frezza C, Cipolat S, Scorrano L (2007) Organelle isolation: functional mitochondria from mouse liver, muscle and cultured fibroblasts. Nat Protoc 2: 287–295. 10.1038/nprot.2006.478 . 17406588

[36] Jagtap P, Soriano FG, Virág L, Liaudet L, Mabley J, Szabó E, et al (2002) Novel phenanthridinone inhibitors of poly (adenosine 5'-diphosphate-ribose) synthetase: potent cytoprotective and antishock agents. Crit Care Med 30: 1071–82. . 12006805 10.1097/00003246-200205000-00019

[37] Chen M, Zsengellér Z, Xiao CY, Szabo C (2004) Mitochondrial-to-nuclear translocation of apoptosis-inducing factor in cardiac myocytes during oxidant stress: potential role of poly(ADP-ribose) polymerase-1. Cardiovasc Res 63: 682–8. . 15306224 10.1016/j.cardiores.2004.04.018

[38] Erdélyi K, Bai P, Kovacs I, Szabo E, Mocsar G, Kakuk A, et al (2009) Dual role of poly(ADP-ribose) glycohydrolase in the regulation of cell death in oxidatively stressed A549 cells. FASEB J 23: 3553–63. 10.1096/fj.09-133264 . 19571039 PMC2747681

[39] Rossi MN, Carbone M, Mostocotto C, Mancone C, Tripodi M, Maione R, et al (2009) Mitochondrial localization of PARP-1 requires interaction with mitofilin and is involved in the maintenance of mitochondrial DNA integrity. J Biol Chem 284: 31616–24. 10.1074/jbc.M109.025882 . 19762472 PMC2797232

[40] Szczesny B, Brunyanszki A, Olah G, Mitra S, Szabo C (2014) Opposing roles of mitochondrial and nuclear PARP1 in the regulation of mitochondrial and nuclear DNA integrity: implications for the regulation of mitochondrial function. Nucleic Acids Res 42: 13161–73. 10.1093/nar/gku1089 . 25378300 PMC4245951

[41] Zhang P, Wong C, Liu D, Finegold M, Harper JW, Elledge SJ (1999) p21(CIP1) and p57(KIP2) control muscle differentiation at the myogenin step. Genes Dev 13: 213–24. . 9925645 10.1101/gad.13.2.213 PMC316389

[42] Kraus WL (2008) Transcriptional control by PARP-1: chromatin modulation, enhancer-binding, coregulation, and insulation. Curr Opin Cell Biol 20: 294–302. 10.1016/j.ceb.2008.03.006 . 18450439 PMC2518631

[43] Andrabi SA, Umanah GK, Chang C, Stevens DA, Karuppagounder SS, Gagné JP, et al (2014) Poly(ADP-ribose) polymerase-dependent energy depletion occurs through inhibition of glycolysis. Proc Natl Acad Sci U S A 111: 10209–14. 10.1073/pnas.1405158111 . 24987120 PMC4104885

[44] Eliasson MJ, Sampei K, Mandir AS, Hurn PD, Traystman RJ, Bao J, et al (1997) Poly(ADP-ribose) polymerase gene disruption renders mice resistant to cerebral ischemia. Nat Med 3: 1089–95. . 9334719 10.1038/nm1097-1089

[45] Heller B, Wang ZQ, Wagner EF, Radons J, Bürkle A, Fehsel K, et al (1995) Inactivation of the poly(ADP-ribose) polymerase gene affects oxygen radical and nitric oxide toxicity in islet cells. J Biol Chem 270: 11176–80. . 7744749 10.1074/jbc.270.19.11176

[46] Pieper AA, Walles T, Wei G, Clements EE, Verma A, Snyder SH, et al (2000) Myocardial postischemic injury is reduced by polyADPribose polymerase-1 gene disruption. Mol Med 6: 271–82. . 10949908 PMC1949947

[47] Ding Y, Zhou Y, Lai Q, Li J, Gordon V, Diaz FG (2001) Long-term neuroprotective effect of inhibiting poly(ADP-ribose) polymerase in rats with middle cerebral artery occlusion using a behavioral assessment. Brain Res 915: 210–7. . 11595210 10.1016/s0006-8993(01)02852-9

[48] Bai P, Cantó C, Oudart H, Brunyánszki A, Cen Y, Thomas C, et al (2011) PARP-1 inhibition increases mitochondrial metabolism through SIRT1 activation. Cell Metab 13: 461–8. 10.1016/j.cmet.2011.03.004 . 21459330 PMC3086520

[49] Kauppinen A1, Suuronen T, Ojala J, Kaarniranta K, Salminen A (2013) Antagonistic crosstalk between NF-κB and SIRT1 in the regulation of inflammation and metabolic disorders. Cell Signal 25: 1939–48. 10.1016/j.cellsig.2013.06.007 . 23770291

[50] Koch-Nolte F, Fischer S, Haag F, Ziegler M (2011) Compartmentation of NAD^+^-dependent signalling. FEBS Lett 585: 1651–6. 10.1016/j.febslet.2011.03.045 . 21443875

[51] Dölle C, Rack JG, Ziegler M (2013) NAD^+^ and ADP-ribose metabolism in mitochondria. FEBS J 280: 3530–41. 10.1111/febs.12304 . 23617329

[52] Frederick DW, Davis JG, Dávila A Jr, Agarwal B, Michan S, Puchowicz MA, et al (2014) Increasing NAD^+^ synthesis in muscle via nicotinamide phosphoribosyltransferase is not sufficient to promote oxidative metabolism. J Biol Chem 290: 1546–58. 10.1074/jbc.M114.579565 . 25411251 PMC4340401

[53] Pirinen E, Cantó C, Jo YS, Morato L, Zhang H, Menzies KJ, et al (2014) Pharmacological inhibition of poly(ADP-ribose) polymerases improves fitness and mitochondrial function in skeletal muscle. Cell Metab 19: 1034–41. 10.1016/j.cmet.2014.04.002 . 24814482 PMC4047186

[54] Mohamed JS, Hajira A, Pardo PS, Boriek AM (2014) MicroRNA-149 inhibits PARP-2 and promotes mitochondrial biogenesis via SIRT-1/PGC-1α network in skeletal muscle. Diabetes 63: 1546–59. 10.2337/db13-1364 . 24757201

